# Liquid Air

**Published:** 1896-03

**Authors:** 


### Liquid Air.

Professor Dewar has exhibited at the Royal Institution the working of a new apparatus for the production of liquid air with a degree of ease not hiherto attainable. Around a cylindrical vacuum-jacketed vessel Professor Dewar closely coils a metallic tube. This is inserted into a second vacuum-jacketed vessel, the result being that the metal tube is protected from external heat by a vacuum both inside and outside the coil. The inner end of the tube has a pinhole orifice which acts as a stopcock, and the outer end is connected to a bottle of condensed air at a pressure of, say 200 atmospheres. On opening the stopcock of the air reservoir, the condensed air passing through the coil to the bottom of the outer vacuum vessel is enormously cooled by expansion on passing the pinhole. It has no mode of escape, save by forcing its way upward between the metallic coil and the glass walls which surround it outside and in. By its passage the coil is powerfully cooled and the condensed air passing through it reaches the nozzle at a lower temperature than before. After this proceess has been carried on for a few minutes liquid air makes its appearance at the nozzle and collects in the outer vacuum vessel, where, in a few minutes more, quantities of 70 or 80 c c, can be obtained with ease. The process is facilitated by cooling the condensed air on its way to the coil, as by passing the tube through solid carbonic acid. With this refinement liquid air appears in three or four minutes, and collects with great rapidity. The new apparatus does not appreciably reduce the heavy expense incident to experiments at low temperatures.

